# Targeting annexin A2 reduces tumorigenesis and therapeutic resistance of nasopharyngeal carcinoma

**DOI:** 10.18632/oncotarget.4521

**Published:** 2015-07-09

**Authors:** Chang-Yu Chen, Yung-Song Lin, Chi-Long Chen, Pin-Zhir Chao, Jeng-Fong Chiou, Chia-Chun Kuo, Fei-Peng Lee, Yung-Feng Lin, Yu-Hsuan Sung, Yun-Tien Lin, Chang-Fan Li, Yin-Ju Chen, Chien-Ho Chen

**Affiliations:** ^1^ School of Medical Laboratory Science and Biotechnology, College of Medical Science and Technology, Taipei Medical University, Taipei, Taiwan; ^2^ Department of Otolaryngology, School of Medicine, College of Medicine, Taipei Medical University, Taipei, Taiwan; ^3^ Department of Otolaryngology, Chi Mei Medical Center, Tainan, Taiwan; ^4^ Department of Pathology, Taipei Medical University Hospital; Department of Pathology, School of Medicine, College of Medicine, Taipei Medical University, Taipei, Taiwan; ^5^ Department of Otolaryngology, Shuang Ho Hospital, New Taipei City, Taiwan; ^6^ Department of Radiation Oncology, Taipei Medical University Hospital, Taipei, Taiwan; ^7^ Department of Radiology, School of Medicine, College of Medicine, Taipei Medical University, Taipei, Taiwan; ^8^ Cancer Center, Taipei Medical University Hospital, Taipei, Taiwan; ^9^ Department of Otolaryngology, Head and Neck Surgery, Wan-Fang Hospital, Taipei, Taiwan; ^10^ Graduate Institute of Biomedical Materials and Engineering, College of Oral Medicine, Taipei Medical University, Taipei, Taiwan

**Keywords:** nasopharyngeal carcinoma (NPC), annexin A2 (ANXA2), chemotherapy, radiotherapy, epithelial-mesenchymal transition (EMT)

## Abstract

The expression of annexin A2 (ANXA2) in nasopharyngeal carcinoma (NPC) cells induces the immunosuppressive response in dendritic cells; however, the oncogenic effect and clinical significance of ANXA2 have not been fully investigated in NPC cells. Immunohistochemical staining for ANXA2 was performed in 61 patients and the association with clinicopathological status was determined. Short hairpin (sh)RNA knockdown of ANXA2 was used to examine cellular effects of ANXA2, by investigating alterations in cell proliferation, migration, invasion, adhesion, tube-formation assay, and chemo- and radiosensitivity assays were performed. RT-qPCR, Western blotting, and immunofluorescence were applied to determine molecular expression levels. Clinical association studies showed that the expression of ANXA2 was significantly correlated with metastasis (*p* = 0.0326) and poor survival (*p* = 0.0256). Silencing of ANXA2 suppressed the abilities of cell proliferation, adhesion, migration, invasion, and vascular formation in NPC cell. ANXA2 up-regulated epithelial-mesenchymal transition associated signal proteins. Moreover, ANXA2 reduced sensitivities to irradiation and chemotherapeutic drugs. These results define ANXA2 as a novel prognostic factor for malignant processes, and it can serve as a molecular target of therapeutic interventions for NPC.

## INTRODUCTION

Nasopharyngeal carcinoma (NPC) is the most common type of malignant tumor that occurs at the nasopharynx. As one of the most common cancers among people of south-eastern Chinese and Asian ancestry, it poses serious health problems in Asia [1, 2]. Characteristically, NPC differs from other head and neck carcinomas in terms of epidemiology, clinical presentations, biological markers, carcinogenic risk factors, prognostic factors, and treatment, and also in its near consistent association with the Epstein-Barr virus (EBV) and a high incidence of metastasis [3, 4]. Currently available clinical therapies for NPC are radiotherapy, chemotherapy, and a combination of the two. Although there is a good efficiency of cure with early-stage NPC patients using radiotherapy (RT) alone, those in the late stage and with distant metastatic spread are difficult to cure [5]. Recently, immunotherapy has been extensively studied in NPC, and immunotherapeutic strategies focused on EBV-induced NPC using an adenoviral vector-based vaccine [6] or EBV-specific cytotoxic T lymphocytes (EBV-CTLs) to suppress tumor progression [7, 8]. Besides CTLs, other studies also indicated the functions of dendritic cells (DCs) in cancer immunotherapy such as the tumor infiltrating DCs reflect on the prognosis and survival in NPC [9–11]. Through NPC cells and DCs interaction, DC functions are also inhibited by a ligand, called annexin A2 (ANXA2), which allows NPC cells to escape from immune surveillance [12]. An immunosuppressive reaction might be the main way through which NPC escapes from attack by immune cells. Therefore, we propose that ANXA2 may be a therapeutic target in NPC.

Annexins constitute a family of at least 20 different calcium-dependent phospholipid-binding proteins that up-regulating cellular growth and signal transduction pathways. ANXA2 exists as both a monomer and a bivalent heterotetramer. The membrane form of ANXA2 is a heterotetramer (two molecules each of annexin A2 and S100A10). S100A10, also called p11, combines with plasminogen. Plasminogen can be cleaved into plasmin, which activates pro-matrix metalloproteases (MMPs) into active MMPs. MMPs are associated with the invasive ability by degrading the cell matrix. The cytosolic form of ANXA2 is a monomer. The ANXA2 monomer participates in some molecular mechanisms when it is phosphorylated and enters nuclei [13]. ANXA2 has been proven to stimulate the proliferation, invasion, and metastasis of cancers, including ovarian, hepatocellular, glioma, pancreatic, and breast cancers [14–18]. However, the knowledge of functions of ANXA2 in NPC is very limited.

In the present study, we investigated the tumorigenic functions of ANXA2 in NPC. We found that silencing ANXA2 reduced various malignant phenotypes as demonstrated by cell proliferative, migratory, invasive, and epithelial-mesenchymal transition (EMT) properties, and increased chemo- and radiosensitivity. The potential effects of ANXA2-knockdown in xenograft tumor-bearing mice and the significance of the clinical pathology were evaluated. Results of these experiments revealed that ANXA2 provides a foundation for studying potential clinical applications in the prognosis or as a therapeutic target in NPC.

## RESULTS

### Clinicopathologic correlations with ANXA2 expression

**Figure 1:**
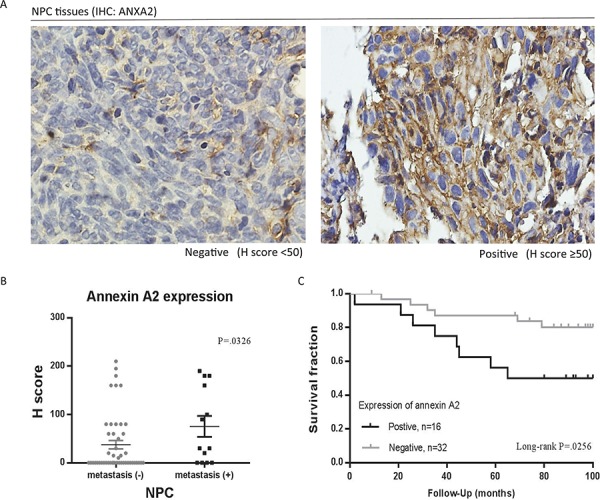
Association of annexin A2 (ANXA2) with clinicopathological variables in nasopharyngeal carcinoma (NPC) **A.** Immunohistochemical (IHC) staining for ANXA2 in tissues of patients. 50 points of H-score was set as the cut off value for determination of positive/ negative. **B.** NPC tissues with metastasis (+) had higher expression of ANXA2. **C.** The overall survival of primary NPC patients (*n* = 48) with positive expression of ANXA2 were significantly shorter than patients with negative expression of ANXA2. The difference in survival was statistically significant (*P* = 0.0256) by log-rank test. *p* < 0.05, there was a significant difference between positive and negative group by log-rank test. The expression level was calculated by H scoring. Results are presented as mean ± SEM. *p* < 0.05, significantly differ from the control by *t*-test.

To investigate the clinical significance of ANXA2, specimens from 61 patients with NPC were obtained for this study. ANXA2 expression levels were examined by IHC and measured by H-score. As to the expression of ANXA2 in NPC, 22 (36.06%) of 61 primary specimens were stained positively for ANXA2. A representative example of different staining results of ANXA2 is shown in Figure 1A. Determination of positive or negative reaction was based on whether H-score was higher or lower than 50 point. The statistical significance of ANXA2 was analyzed in regard to the clinical data, including age, sex, histologic types by Chi-squared test. There were no statistical correlations of ANXA2 levels with gender (*p* = 0.811) or age (*p* = 0.871), but there were statistical correlations with metastasis (*p* = 0.0326) (Figure 1B). The association of ANXA2 expression and patient overall survival was examined using the Kaplan-Meier method with a log-rank test. As shown in Figure 1C, positive of ANXA2 was associated with a poor prognosis (*p* = 0.0256), in primary NPC patients.

### Knockdown of ANXA2 inhibits cell proliferation in NPC cell lines

**Figure 2:**
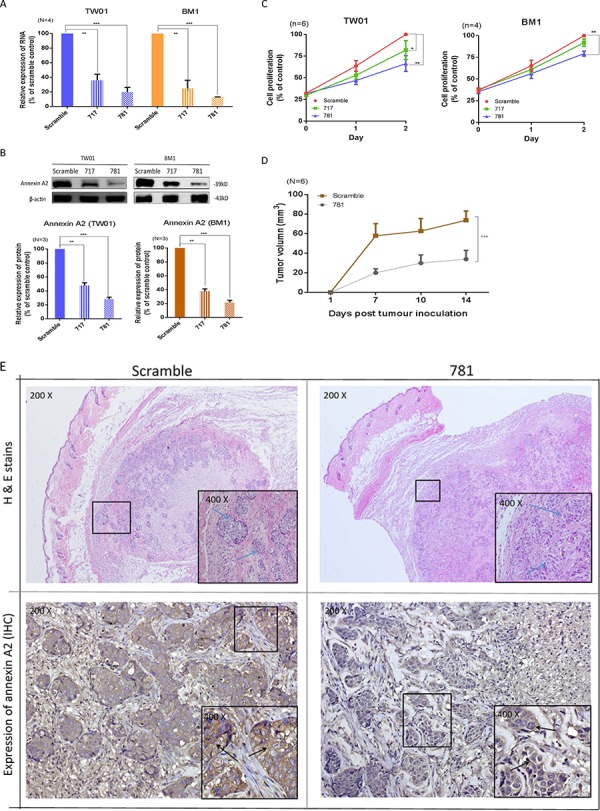
Silencing of annexin A2 (ANXA2) ihibits cell proliferation both *in vitro* and *in vivo* ANXA2 expression was suppressed by shRNA. TW01 and BM1 cells were transduced with two ANXA2-specific shRNA sequences (717 and 781) or a scrambled control, and stable clones were selected by puromycin. **A.** A real-time PCR was used to determine ANXA2 mRNA expression levels in TW01 and BM1 cells. GAPDH was used as an internal control. **B.** Western blotting was used to determine the ANXA2 protein expression level. β-Actin served as an internal control. Semiquantitative results of the Western blot assay were measured by UN-scan-it software. Results are presented as the mean ± SEM of triplicate determinations from three independent experiments. Knockdown of ANXA2 expression reduced the proliferation of nasopharyngeal carcinoma (NPC) cells: **C.** TW01 and BM1 cells. Cell proliferation was detected on days 1 and 2 by an MTT assay. Results are presented as the mean ± SEM of triplicate determinations from six independent experiments. In *in vivo* studies, ANXA2 silencing suppressed the growth of xenograft tumors. **D.** Six NOD.CB17-Prkdcscid/JNarl mice in each group were subcutaneously injected with 2 × 10^6^ TW01-scrambled or TW01-781 cells. The tumor size was calculated as (S^2^ × L)/2 (S, short diameter; L, long diameter). Results are presented as the mean ± SEM. *p < 0.05; **p < 0.01; ***p < 0.001, significantly differs from the control (by a *t*-test). **E.** Tissues of xenograft tumors, scrambled and 781, were confirmed by H&E staining, and also immunohistochemical staining for ANXA2.

To evaluate the cellular functions of ANXA2, two stable ANXA2-specific knockdown cell lines were established after transduction of shRNA targeting ANXA2 into the TW01 and BM1 NPC cell lines. Messenger (m)RNA expressions of TW01-717 and TW01-781 were respectively reduced 70% and 86%, while those of BM1- 717 and BM1-781 were respectively reduced 75% and 87% (Figure 2A). Protein expression levels of TW01-717 and TW01-781 were respectively reduced 50% and 64%, while those of BM1-717 and BM1-781 were respectively reduced 70% and 80% (Figure 2B). The efficiencies of shRNA knockdown were similar between the TW01 and BM1 cell lines. Stable ANXA2-knockdown cells were used in subsequent cellular studies. Silencing of ANXA2 suppressed cell proliferation in TW01-717 and 781 cells by 78% and 63%, respectively, on day 2, and similar effects were also observed in BM1 cells (Figure 2C). Our data suggested that suppression of ANXA2 could reduce cell proliferation in both NPC cell lines. To investigate the effects of ANXA2 knockdown on tumor growth *in vivo*, we established xenograft NPC tumors in NOD/SCID mice. ANXA2-knockdown NPC in mice showed significant growth arrest compared to the controls. On average, the 781 cell group exhibited 50% decreased tumor growth on day 21 (*p* = 0.001) (Figure 2D). Tumor tissues were confirmed by H&E staining (Figure 2E), and to verify that the tumor growth rate was influenced by ANXA2 knockdown, the xenograft tumors were dissected to examine ANXA2 expression by IHC. As shown in Figure 2E, the ANXA2 protein was reduced in the 781 cell group compared to the scrambled control group. The results indicated that ANXA2 promoted tumor cell proliferation both *in vivo* and *in vivo* and suggested that ANXA2 could be a molecular target for treatments aimed at decreasing oncogenesis.

### ANXA2 silencing suppresses cell migration, invasion, vascular formation, and cell adhesion of NPC

**Figure 3:**
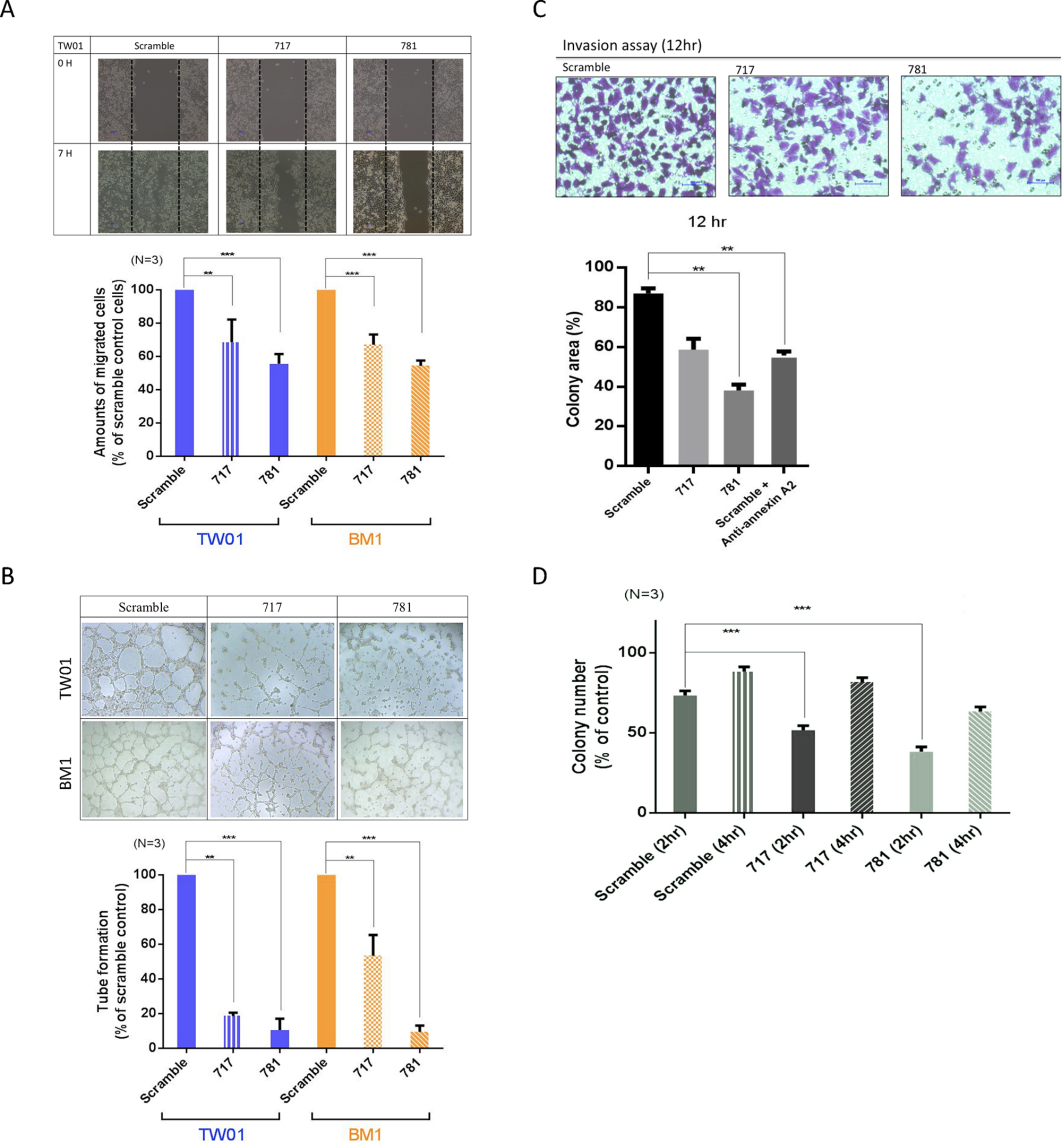
Annexin A2 (ANXA2) knockdown inhibits malignant phenotypes *in vitro* Knockdown of ANXA2 suppressed cell migration and invasion, vascular formation, and the adhesive ability. **A.** The cell-migratory ability was reduced in ANXA2-knockdown cells, as determined by a wound-healing assay. Migration assay data were quantitatively analyzed. **B.** Tube formation assay and quantitative data for testing the vascular formation ability. **C.** The cell invasive ability was suppressed in 781 and 717 cells, as determined by a Matrigel invasion assay. Numbers of cells that had migrated through the Matrigel to the bottom of the chamber were determined at 12 h. **D.** Adhesion assay for the TW01 cell line (scrambled, 717, and 781). Quantitative analytical data were compared to the scrambled control and reported as a percentage. Results are presented as the mean ± SEM, *n* = 3. *p < 0.05; **p < 0.01; ***p < 0.001, significantly differs from the control (by a *t*-test).

In advanced stages of NPC, the major cause of treatment failure is distant metastasis. The potential effects of ANXA2 on cell mobility and invasion were examined in stable ANXA2-knockdown cells. Compared to the scrambled control, the cell migration ability decreased 70% (717) and 55% (781) in TW01 cells, and the same effect was also observed in BM1 cells (Figure 3A). The vascular formation ability decreased to 20% (717) and 15% (781) in TW01 cells, and 60% (717) and 10% (781) in BM1 cells (Figure 3B). Cell invasion results from the Matrigel assay are shown in Figure 3C. The invasive abilities decreased 50% in 717 cells and 75% in 781 cells after 12 h compared to scrambled control cells (*p* < 0.01). Moreover, an anti-ANXA2 antibody also suppressed the cell invasive ability (Figure 3C). Lastly, we also observed whether ANXA2 regulates the cell adhesive ability in NPC. After 2 h of cell culture, 75% of scrambled cells had attached to the well. However, only 50% of 717 cells had, and < 25% of 781 cells had (*p* = 0.01). The results suggest that ANXA2 knockdown inhibited the cell adhesion function (Figure 3D). These results demonstrate that silencing of ANXA2 suppressed cell migration, invasion, vascular formation, and cell adhesion abilities.

### Expression of ANXA2 induces the EMT by activating the transforming growth factor (TGF)-β pathway

**Figure 4:**
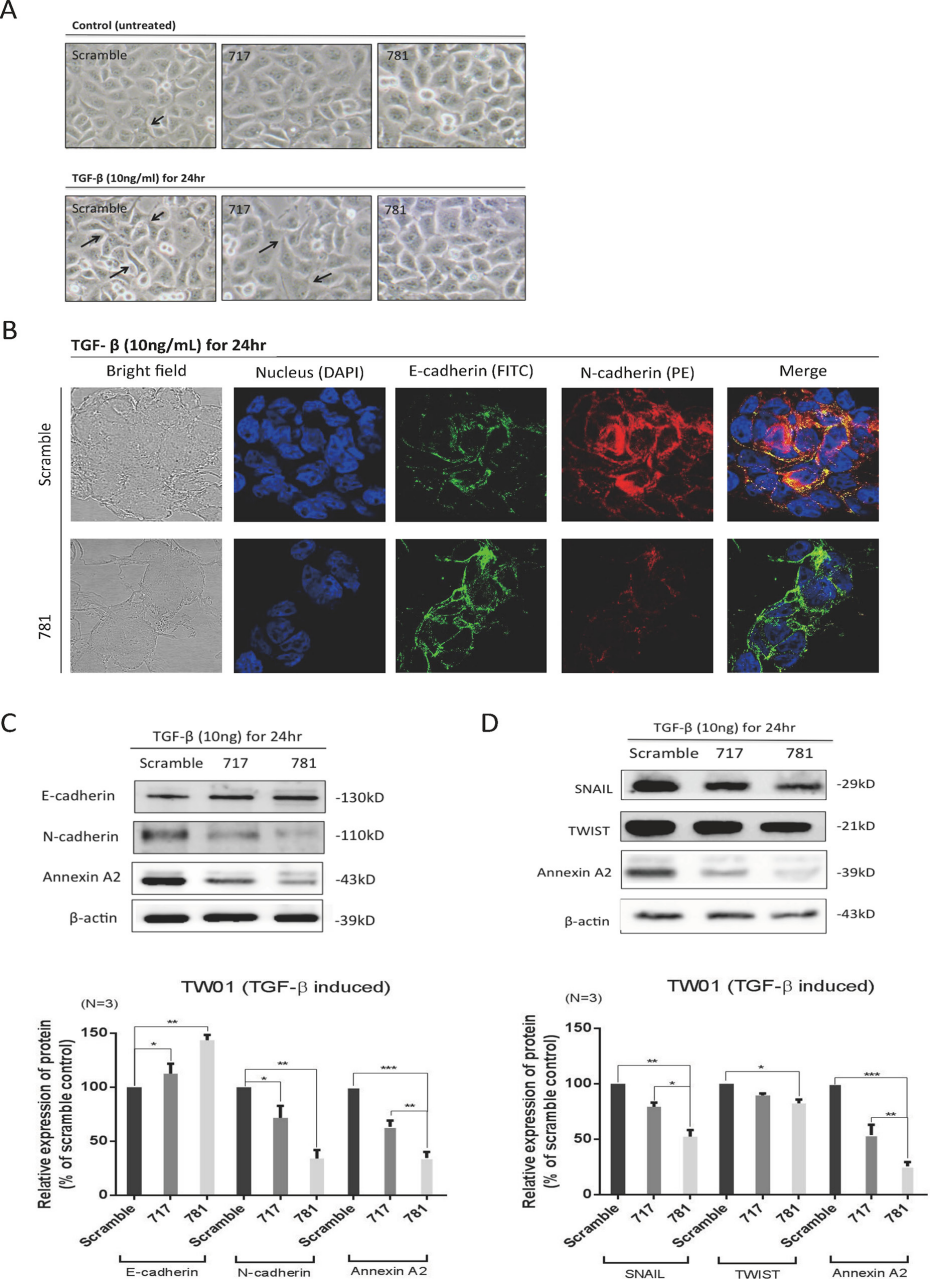
Annexin A2 (ANXA2) regulates the epithelial-mesenchymal transition (EMT) in nasopharyngeal carcinoma (NPC) cells **A.** Cell images with and without transforming growth factor (TGF)-β treatment. The cell morphology slightly changed when treated with TGF-β, as it became more spread out compared to untreated cells in scrambled, sh717, and sh781 cells. The arrows indicate morphological changes in mesenchymal-like cells. Silencing of ANXA2 regulated EMT marker molecules. **B.** Immunofluorescence, confocal microscopy, and **C.** Western blotting were used to examine expression levels of E-cadherin and N-cadherin in sh781 and scrambled control cells. Semiquantitative Western blot data were analyzed. **D.** Western blotting for EMT signal proteins: SNAIL and TWIST. Semiquantitative data from Western blotting are presented as the mean ± SEM of triplicate determinations (*n* = 3). *p < 0.05; ** *p* < 0.01; ***p < 0.001, significantly differs from the control (by a *t*-test).

Since silencing of ANXA2 reduced cell migration, invasion, and adhesion abilities, we therefore investigated whether ANXA2 also participated in the EMT process in NPC cells. We used TGF-β, known as an EMT stimulus, to reinforce the transition speed. Indeed, scrambled cells (with high ANXA2 expression) exhibited increased cell scattering when treated with 10 ng/ml TGF-β for 24 h (Figure 4A). E-cadherin and N-cadherin expression levels were confirmed by immunofluorescence. Higher levels of the mesenchymal marker, N-cadherin, and lower levels of the epithelial marker, E-cadherin, were expressed in scrambled control cells, but the phenomenon was reversed after silencing of ANXA2 (Figure 4B). Moreover, Western blotting confirmed that E-cadherin and N-cadherin expression levels showed consistent results (Figure 4C). In terms of the EMT signal pathway, silencing of ANXA2 produced a decrease in TGF-β-induced Snail/Twist expression level in NPC cells (Figure 4D).

### Knockdown of ANXA2 increases radio- and chemosensitivity

**Figure 5:**
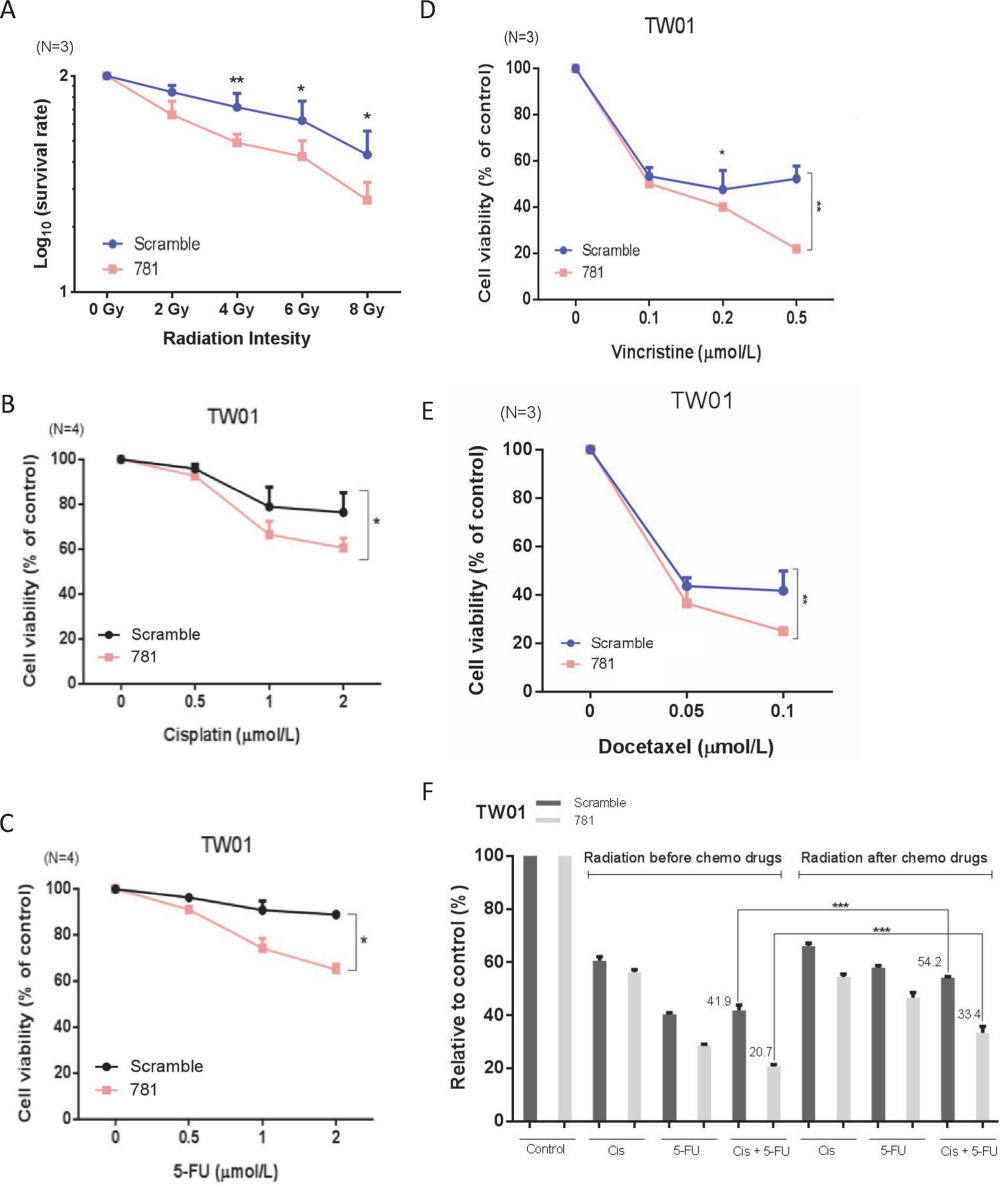
Silencing of annexin A2 (ANXA2) increases radio- and chemosensitivity *in vitro* Irradiation resistance was determined in TW01-781 and TW01-scrambled cells by a clonogenic survival assay after treatment with various doses (0~8 Gy) of radiation **A.** Resistance to chemotherapeutic drugs (cisplatin, 5-FU, docetaxel, and vincristine) was determined by a cytotoxic assay after treatment of TW01-781 and TW01-scrambled cells with various doses of **B.** cisplatin, **C.** 5-FU, **D.** docetaxel, and **E.** vincristine for 24 h. Cytotoxic assay data were compared to the control (no drugs), and results are presented as the mean ± SEM of triplicate determinations from three independent experiments. **F.** Quantitative analysis of surviving colonies with a combination of chemotherapeutic drugs (cisplatin and 5-FU) and irradiation. Results are presented as the mean ± SEM of triplicate determinations from three independent experiments. *p < 0.05; **p < 0.01; ***p < 0.001, significantly differs from the control (by a *t*-test).

Radiotherapy is the main therapeutic method for NPC. However, the efficiency of radiotherapy in late-stage patients is not good. Chemotherapy is often used together with radiation therapy for more-advanced stages of NPC; therefore, whether ANXA2 increased the radio- and chemosensitivity of NPC cells was examined. For the radiosensitivity assay, cells were treated with various radiation doses (0~8 Gy) and cultured for 7 days to allow colony formation. Silencing of ANXA2 resulted in fewer surviving colonies compared to scrambled control cells, and survival rates were 0.82, .079 and 0.75 at 4, 6, and 8 Gy, respectively (Figure 5A). The chemotherapeutic drugs of cisplatin, 5-FU, docetaxel, and vincristine were used to test for drug sensitivity. Silencing of ANXA2 increased drug sensitivity by 30% and 21%, respectively, after treatment with cisplatin or 5-FU for 24 h (Figure 5B, 5C). When cells were treated with vincristine or docetaxel, cells exhibited 32% and 20% enhanced drug sensitivity after 24 h with a low concentration of drugs (Figure 5D, 5E). Thus, we determined that ANXA2 regulates the therapeutic tolerance of both radio- and chemotherapies in NPC. According to data from irradiation and chemotherapeutic drugs, we tried to determine whether the order of therapy (radiotherapy and chemotherapy) had any difference on the treatment efficiency after silencing of ANXA2. We separated cells into two groups: one was given chemotherapeutic drugs (cisplatin or 5-FU) before irradiation. The other group was given chemotherapeutic drugs after irradiation. The results showed that more NPC cells were killed when NPC cells were exposed to irradiation before treatment with chemotherapeutic drugs after silencing of ANXA2 (Figure 5F). Thus, the order of chemotherapy and radiotherapy is important for treatment effects.

## DISCUSSION

To our knowledge, this is the first report to investigate the potential clinicopathologies, functions and molecular mechanisms of ANXA2 in NPC. ANXA2 is upregulated in several cancers and is associated with metastasis [14–18]. There are very few studies on ANXA2 in NPC so far. A proteomics comparison that analyzed an EBV-associated NPC cell line (C666–1) and normal nasopharyngeal cell line (NP69) showed that ANXA2 was downregulated in the NPC cell line and found that cytoplasmic staining, complete and incomplete membrane staining, and both cytoplasmic and membrane staining were all lost in clinical tissues [19]. However, our studies showed that ANXA2 is associated with the clinicopathological status of NPC from patients’ tissues. Those previous results were opposite to our findings, and we inferred that this might have been due to different types of NPC tissues or the EBV-infection status. We collected different types of NPC tissues, but the previous report had limited samples of an undifferentiated type of NPC tissues. The expression level of ANXA2 was correlated with poor survival, which may reflect that ANXA2 regulates cell proliferation at the cellular level and therapeutic response (Figure 2 and 5). The association with metastasis can be explained by the knockdown of ANXA2 suppressing cell migration, invasion, vascular formation, and adhesion abilities (Figure 3). These findings are in accord with the results of the previous studies that up-regulated ANXA2 correlated with the clinical aggressiveness in renal cancer, endometrial cancer and colorectal carcinoma [20–22]. Meanwhile, a meta-analysis was collected from fifteen studies including 2,321 patients, 14 cancer types (not including NPC) demonstrated that the overexpression of ANXA2 was correlated with poor prognosis in term of overall survival, disease-free survival and significantly associated with tumor invasion and lymph mode metastasis [23]. This result might reveal that ANXA2 might be a biomarker for malignant tumors.

Epithelial– mesenchymal transition (EMT) is important in embryonic development and cancer metastasis. The cellular event during EMT is epithelial cells lose their cell-cell adhesion and cell polarity, and gain migratory and invasive properties to become mesenchymal cells [24]. It has been characterized by down-regulation of epithelial markers such as E-cadherin and up-regulation of mesenchymal markers like N-cadherin, fibronectin, and vimentin [24]. Transcriptional factors such as Twist1, Snail1 Snail 2, ZEB1 and ZEB2 are also governing EMT [25]. This dynamic process could be stimulated by the signals and forms the microenvironment like TGF-β, Wnt, and TNFα [24–26]. In the present study, silencing of ANXA2 suppressed cell mobility (Figure 3) might occur through an EMT process by altering expression of the E-cadherin, N-cadherin, snail and twist after TGF-β stimulated. The membrane form of ANXA2 is a heterotetramer (two molecules each of ANXA2 and two S100A10). S100A10, also called p11, can combine with plasminogen. Plasminogen is cleaved into plasmin, which can activate pro-MMPs into active MMPs. MMP-2 and -9 are associated with the invasive ability by degrading the cell matrix [13]. ANXA2 may participate in extracellular matrix digestion to enable invasive behavior. In our study, we found that knockdown of ANXA2 expression reduced a cell's invasive ability (Figure 3C). Therefore, the reduction in the invasive ability through suppression of MMP activity should be further investigated. To identify the tumor malignancy, the angiogenesis is also one of the consideration factors. Vasculogenic mimicry (VM) is important way for tumor development and angiogenesis [27], and silencing of ANXA2 suppressed the vascular formation ability which indicates that ANXA2 may suppress angiogenesis (Figure 3B). ANXA2 regulates metastasis may proceed through EMT transition, extracellular matrix digestion and angiogenesis pathway.

We confirmed that ANXA2 was correlated with therapeutic resistance (Figure 5), and consistent result was also reported in lung cancer [28]. The potential effect of ANXA2 in therapeutic resistance may due to the enlargeosomes and nuclear ANXA2. ANXA2 coats the surface of enlargeosomes, which regulate exocytosis. Enlargeosomes are widely expressed, non-secretory vesicles, which are involved in Ca^2+^-dependent cell surface trafficking of membranes [29–31]. We thought that knockdown of the ANXA2 protein would influence the number and function of enlargeosomes. If enlargeosomes cannot undergo exocytosis, chemotherapeutic drugs might remain in cells and kill tumor cells more quickly. ANXA2 can mitigate DNA damage from irradiation by entering nuclei to protect the DNA. However, the mechanism of how ANXA2 protects DNA is unclear at present [32]. Furthermore, the role of the annexin family of ANXA1 has been studied during radiotherapy and chemotherapy. The expression level of ANXA1 was associated with drug-resistance in NPC [33]. And, ANXA1 participate in tumor irradiation resistance in NPC via regulating cell apoptosis [34].

Cellular signaling of ANXA2 was reported by Wei Luo et al. [35]. They found that LMP1 increases the serine phosphorylation level of ANXA2 by activating the phosphoinositide-specific phospholipase C (PI-PLC)-protein kinase C (PKC) α/PKC β pathway, which plays an important role in serine phosphorylation and nuclear entry of ANXA2. Serine 25 phosphorylation of ANXA2 was associated with its nuclear entry, DNA synthesis, and cell proliferation. In addition, we found that knockdown of ANXA2 decreased the expression of total Akt and phosphorylation at Ser473 (Supplementary Figure 1). The oncogenic serine/threonine kinase, Akt, a downstream signal protein of phosphatidylinositol 3′ kinase (PI3K), was shown to regulate many kinds of genes and proteins. Through the Akt pathway, Akt is involved in cell proliferation, survival, growth, and other undetected functions [36]. Additional reports claimed that (1) radiation enhances cell invasion with MMP-9 in hepatocellular carcinoma through the PI3K/Akt/NF-κB signal transduction pathway [37]. (2) The chaperon protein Hsp27 interacts with Akt against UV-induced DNA damage and prevents cell apoptosis by inhibiting p21 activation. [38]. (3) Akt regulates exocytosis by controlling the exocytosis vesicle membrane protein of Rab activation through downstream subtracts AS160/TBC1D1 [39, 40]. (4) Akt suppresses transcription of the E-cadherin and activates of SNAIL gene expression causes the conversion of epithelial cells into invasive mesenchymal cells [41]. Thus, ANXA2 facilitates multiple malignant phenotypes by downregulating the expression level of Akt.

Recently, accumulated evidence suggests that a small sub-population of cells within a tumor called cancer stem cells (CSCs) has stem-like properties and the ability to initiate new tumors [42]. CSCs exhibit important properties such as self-renew, differentiation capacity, resistance to chemo/radio-therapy and cancer invasion and metastasis [43, 44]. Akt signaling has been reported participating in CSC maintenance in various cancer types [45–47] and that constitutive activation of Akt promotes CSC resistance to chemotherapy and radiation therapy [48]. Meanwhile, expression of stemness-related transcription factors such as Oct4, Sox2 and Nanog could be regulated by Akt [49–51]. Here, we found that silencing of ANXA2 reduced cancer stem cell formation ability (Supplementary Figure 2A) and expression of CSC associated markers Oct4, Sox2 and Nanog (Supplementary Figure 2B). Taken together, silencing of ANXA2 inactivate Akt pathway and repress NPC cancer stem cells through inhibiting expression of Sox2, Nanog and Oct4.

In 2013, Lokman et al. reported that anti-ANXA2 antibodies significantly blocked ovarian cancer cell invasion in a CAM model and cancer cell peritoneal dissemination in an intraperitoneal xenograft mouse model which were consistent with our findings (Figure 3C) [14]. Based on the following considerations, ANAX2 could serve as a therapeutic target in NPC. First, knockdown ANAX2 directly suppressed oncogenic properties in NPC cells. Second, ANXA2 reduced sensitivities to irradiation and chemotherapeutic drugs (cisplatin, 5-FU, vincristine, and docetaxel). Third, immunosuppression responses in DCs were medicated through DC-SIGN-recognizing ANXA2 of NPC [52].

In conclusion, our findings demonstrate that ANXA2 promotes therapeutic tolerance and multiple malignant phenotypes, including migration, invasion, epithelial-mesenchymal transition, and cancer stemness. ANXA2 expression was significantly correlated with metastasis and poor survival. In the future, ANXA2 might be used as a target for new therapeutic strategies against nasopharyngeal carcinoma.

## MATERIALS AND METHODS

### Cell culture and establishment of stable ANXA2-silenced cells

Two NPC cell lines, TW01 (provided by CH Tsai, National Taiwan University, Taiwan) and BM1 (provided by YS Chang, Chang Gung University, Taiwan), were used [53]. All cell lines were maintained in Dulbecco's modified Eagle medium (DMEM) containing 10% fetal bovine serum (FBS), 100 units/ml penicillin, and 100 μg/ml streptomycin. All cells were grown at 37°C in a humidified incubator containing 5% CO2. The knockdown of ANXA2 in NPC was performed by lentiviral short hairpin (sh)RNA interference. We selected two shRNA (both obtained from the TRC shRNA library) target sequences, (TRCN0000289717, 5′-GCAGGAAATTAACAGAGTCTA-3′ and TRCN0000 289781, 5′-CGGGATGCTTTGAACATTGAA-3′) specific for the human ANXA2 gene for efficient depletion and a scrambled shRNA control (pLAS.Void) with mismatched sequences. The three shRNAs were cloned from the pLKO.1 vector into AgeI-EcoRI sites of the pLKO.1-hPGK-Puro vector. Then, shRNAs were transfected in packaging HEK 293T cells with helper vectors, using the Fugene 6 transfection reagent (Roche, Basel, Switzerland). Medium containing lentiviral particles was harvested, filtered, separated into aliquots, and stored at −80°C. These viruses were used to transduce 5 × 105 cell/well in the presence of 8 μg/ml polybrene (Sigma-Aldrich, St. Louis, MO, USA) in 6-well plates at a multiplicity of infection (MOI) of 3. Transduced cells were selected in DMEM containing 5 μg/ml puromycin (Sigma-Aldrich, St. Louis, MO, USA). Lastly, we established three stable ANXA2-knockdown cell lines: scrambled, 717, and 781.

### Patient tissue samples

This study was approved by the Institutional Review Broad of Human Investigation Committee in Taipei Medical University (TMU-JIRB No. 201312014). A total of 61 formalin-fixed paraffin nasopharyngeal carcinoma (NPC) tissue samples from primary (*n* = 48) and metastatic (*n* = 13) were obtained from TMU Hospital (Taipei, Taiwan). The original diagnosis for each subject was in accordance with the World Health Organization Classification. The patients included 40 males and 21 females with an age range of 25 to 85 years (mean age: 47.44). Biopsies of tumor samples were obtained from each subject before chemotherapy or radiotherapy. Patients with primary NPC received radiotherapy and those with metastatic NPC received radiotherapy and/or chemotherapy for treatment. The median follow-up time was 78.5 months (range: 2 ~ 100 months).

### Western blotting

Confluent cells were collected and lysed in RIPA buffer (Genestar Biotechnology, Taiwan). In total, 30 μg of proteins was separated by 10% sodium dodecylsulfate - polyacrylamide gel electrophoresis (SDS-PAGE) and transferred onto polyvinylidene difluoride (PVDF) membranes (Life Science, Carlsbad, CA, USA). After blocking, the membranes were hybridized with specific primary antibodies followed by incubation with secondary antibodies conjugated to horseradish peroxidase (HRP). The protein images were detected using the Western Blotting Plus Chemiluminescence Reagent (Thermo Scientific, Waltham, MA, USA). The density of each protein band was determined after normalization to an actin control band using the Gel Image System and Image J software (Scion Corporation, Torrance, MD, USA). The primary antibodies used included anti-ANXA2 (R&D System, Minneapolis, MN USA), anti-Akt, anti- Akt S473p, anti-Akt T308p, anti-Snail, anti-TWIST, anti-E-cadherin, and anti-N-cadherin (all from Genetex, Irvine, CA, USA). Protein images were detected using Western Blotting Plus Chemiluminescence Reagent (Thermo Scientific, Waltham, MA, USA). The density of each protein band was determined after normalization to an actin control band using the Gel Image System and Image J software.

### Real-time polymerase chain reaction (PCR)

Total RNA (1 μg) was reverse-transcribed using oligo (dT) primers and the SuperScript^®^ III First-Strand Synthesis System (Invitrogen, Carlsbad, CA, USA). Quantitative (q)PCRs were performed with FAST STBR^®^ Green Master Mix according to the manufacturer's protocol and performed on an ABi Step One Real-Time PCR System (ABI, Foster City, CA, USA). Sequences of oligonucleotides used for the real-time PCR analysis were ANXA2 (forward primer 5′-CTC TAC ACC CCC AAG TGC A-3′ and reverse primer 5′-TCA GTG CTG ATG CAA GTT CC-3′) and GAPDH (forward primer 5′-CGG AGT CAA CGG ATT TGG TCG TAT G-3′ and reverse primer 5′-AGC CTT CTC CAT GGT GGT GAA GA-3′). GAPDH expression was used as a control to normalize data of other RNA levels.

### Cell proliferation, chemosensitivity, and radiosensitivity assays

In total, 2000 cells with stable ANXA2-silenced or scrambled control cells were seeded in 96-well plates. Cell proliferation was detected at 24 and 48 h by an MTT assay. To determine the chemosensitivity, 2000 cells were seeded for 16 h and treated with various doses of cisplatin, 5-FU, vincristine, and docetaxel. Numbers of surviving cells were counted and compared to numbers of surviving untreated cells. To determine the radiosensitivity, 104 cells were plated in 6-cm dishes, and after incubation for 24 h, cells were exposed to a range of radiation doses (0~8 Gray). After irradiation, cells were further cultured for 7 days. Numbers of surviving colonies (defined as a colony with ≥ 50 cells) were counted. The survival fraction was calculated as the number of colonies divided by the number of cells of the control times 100. The colony number was counted with Image J software.

### Migration, invasion, and adhesion assays

The cell migration assay was performed using an *in vitro* wound healing assay. Cells were seeded in ibidi Culture-Inserts (ibidi, Verona, WI, USA) and incubated until fully confluent; then, culture inserts were detached to form a cell-free gap in a monolayer of cells. The cell migration status toward the gap area was photographed every 7 h. The cell invasion assay was performed using the BD BioCoat Matrigel invasion chambers (Becton Dickinson Biosciences, Bedford, MA, USA) and Millicell invasion chambers (Merck Millipore, Germany). The Millicell upper chamber inserts, the membranes of which have a pore size of 8 mm, were placed in a 24-well plate and coated with Matrigel. Cells were seeded in the upper chambers with media containing 1% FBS. The lower chambers contained complete culture media (10% FBS) to trap the invading cells. The invasive ability of cells was determined at 6 and 12 h. Cells invading the lower side of membrane were fixed, stained with crystal violet, and photographed. For the adhesion assay, plates were coated with collagen I, removed after 12 h, and then air-dried. Cells were seeded onto the plates and incubated for 2 and 4 h. After incubation, unattached cells were washed off with phosphate-buffered saline (PBS), and then cells were fixed and stained with crystal violet and photographed.

### Tube formation assay

Angiogenesis was evaluated by tube formation. Cells of the scrambled control and two strains of shANXA2 (2 × 104 cells/well) were grown in a 96-well plate pre-coated with Matrigel, and incubated for 16 h at 37°C. The formation of capillary-like structures was captured. The degree of angiogenesis was quantified as the number of branching points per view.

### Immunofluorescence staining

Cells were incubated on an EZ slide (Merck Millipore, Germany) overnight, and then cells were fixed with paraformaldehyde and blocked with FBS. Slides were incubated with E-cadherin and N-cadherin antibodies, and stained with an FITC or PE secondary antibody. Samples were mounted with mounting media containing DAPI. The fluorescence was visualized using a confocal laser microscope.

### Xenograft tumor mice model

NOD.CB17-Prkdcscid/JNarl mice were purchased from National Applied Research Laboratories (NARLabs, Taipei, Taiwan). All studies were conducted in accordance with IACUC-approved protocols. In total, 12 mice at 6 weeks of age were used. In total, 2 × 106 TW01 scrambled cells or 781 cells were subcutaneously injected into each mouse. The tumor volume was calculated using the formula (S2 × L)/2, where S is the short length of the tumor and L is the long length of the tumor.

### Immunohistochemistry (IHC)

Formalin-fixed and paraffin-embedded xenograft tumors or NPC tissues were deparaffinized in xylene, boiled in citrate buffer to retrieve the antigens, and blocked with hydrogen peroxide. Slides were incubated with an anti-annexin A2 antibody (R&D system, Minneapolis, MN USA) followed by secondary antibody-conjugated HRP. The reaction was developed using the substrate DAB chromogen system. Counterstaining was performed with hematoxylin before dehydration and mounting. Staining reactions were determined by microscopic examination. Each sample was given a score according to the H-score. H-score = I × P. “ I “ means staining intensity. Score 0, no staining; score 1+, weak staining; score 2+, moderate staining; score 3+, strong staining. “ P “ means percent of cell at each staining intensity level. An individual ANXA2 IHC score (H-score) value from 0 to 300 was generated from the tumor sample of each patient. 50 points of H-score was set as the cut off value for determination of positive/ negative. Positive immunostaining of ANXA2 in lesion tissues and its associations with clinicopathological parameters were analyzed.

### Statistical analysis

All results are expressed as the mean ± standard error of the mean (SEM). Data were compared using a *t*-test. All graphs were drawn using GraphPad Prism 6.0 (GraphPad Software, Inc. La Jolla, CA, USA). A Chi-squared test was used to examine the association of ANXA2 protein expressions and clinicopathologic features. Survival curves were calculated by a log-rank test. All *p* values were 2-sided, and the significance level was set at *p* < 0.05.

## SUPPLEMENTARY FIGURES



## Abbreviations

ANXA2: annexin A2
NPC: nasopharyngeal carcinoma
shRNA: short hairpin ribonucleic acid
EMT: epithelial-mesenchymal transition
TGF-β: transforming growth factor beta.
