# Supplementary material for: Targeting annexin A2 reduces tumorigenesis and therapeutic resistance of nasopharyngeal carcinoma
Source: Oncotarget. 2015 Jul 9;6(29):26946–59. doi: 10.18632/oncotarget.4521 (PMC4694965; doi:10.18632/oncotarget.4521)
Supplement: Supplementary file 1 [file oncotarget-06-26946-s001.pdf]

## SUPPLEMENTARY FIGURES

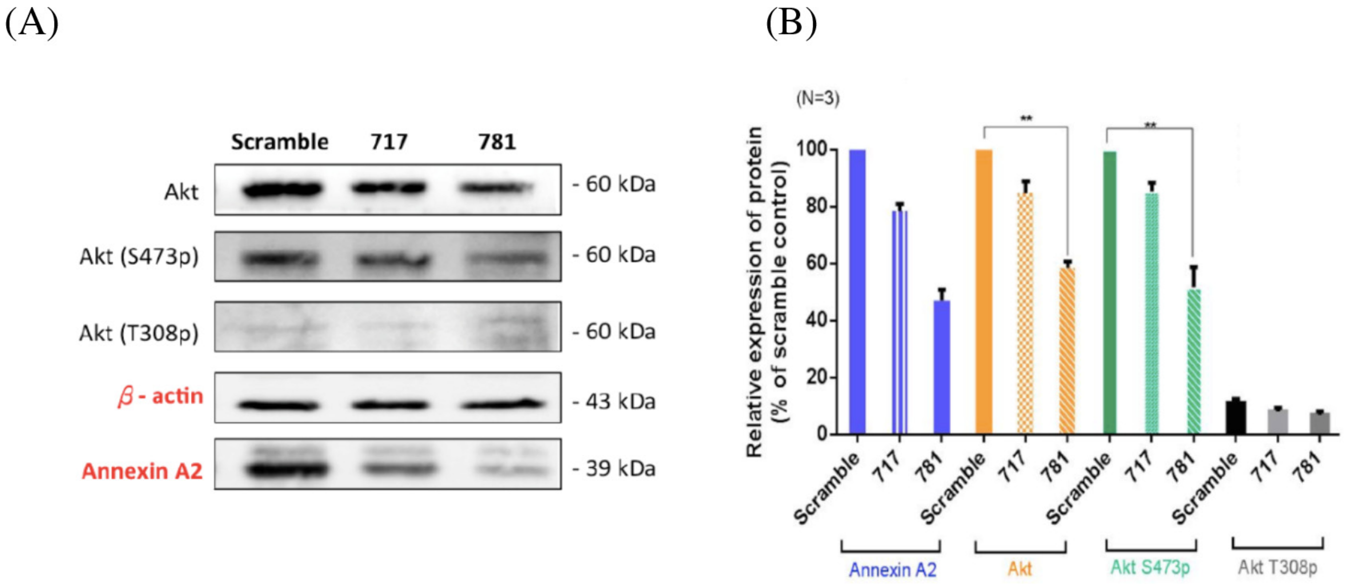


**Supplementary Figure S1: Knockdown of annexin A2 down-regulates the expression of total Akt and Akt phosphorylation at S473. A.** TW01-scramble, TW01–717 and TW01–781 total cell lysates were prepared, and the level of total Akt, Akt phosphorylated at S473 and T308 were assayed by Western blotting. **B.** Semi-quantitative analysis of western blotting, mean ± SEM of triplicates from 3 independent experiments. Mean ± SEM $*P < 0.05$; $**P < 0.01$; $***P < 0.001$, significant different from control (*t*-test).

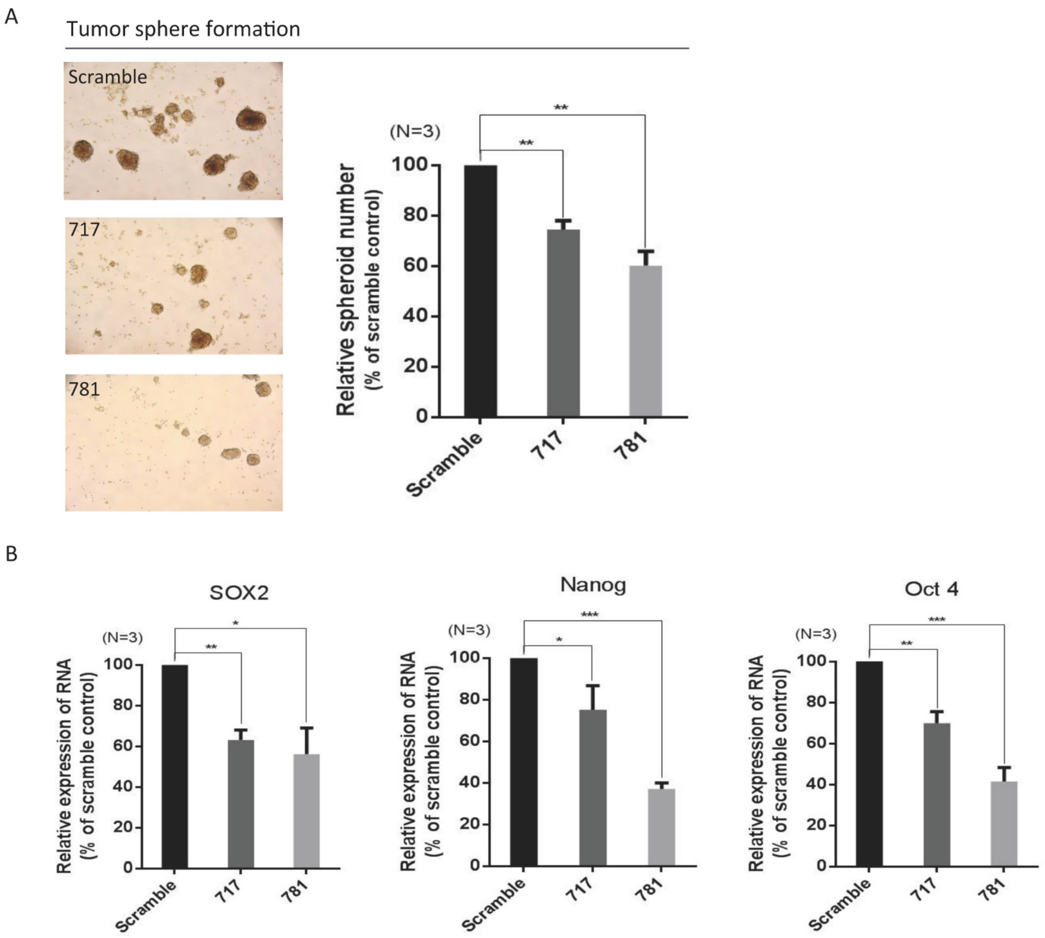


**Supplementary Figure S2: Knockdown of annexin A2 suppressed cancer stemness. A.** Knockdown ANXA2 suppressed tumor sphere formation. TW01-scramble, TW01–717 and TW01–781 cells were cultured in DMEM/F12 serum free medium containing basic fibroblast growth factor (bFGF), epidermal growth factor (EGF) and B27 supplement in ultralow attachment plates. After 2 weeks, tumor spheres were visualized and enumerated by microscopy. **B.** The mRNA levels of Sox2, Oct4 and Nanog in TW01-scramble, TW01–717 and TW01–781 cells were measured by RT-qPCR. *$P < 0.05$, **$P < 0.01$, ***$P < 0.001$. Error bars represent mean ± SD of triplicates.
